# Advancements in Total Knee Arthroplasty over the Last Two Decades

**DOI:** 10.3390/jcm14155375

**Published:** 2025-07-30

**Authors:** Jakub Zimnoch, Piotr Syrówka, Beata Tarnacka

**Affiliations:** 1 Department of Rheumo-Orthopeadics, National Institute of Geriatrics, Rheumatology and Rehabilitation in Warsaw, Spartańska 1, 02-637 Warsaw, Poland; klinika.reumoortopedii@archiwum.spartanska.pl; 2 Department of Rehabilitation, Medical University of Warsaw, Spartańska 1, 02-637 Warsaw, Poland; beata.tarnacka@wum.edu.pl

**Keywords:** total knee arthroplasty, TKA, robotic TKA, cementless implants, 3D-printed knee implants, minimally invasive knee surgery, MIS, rehabilitation, ERAS

## Abstract

Total knee arthroplasty is an extensive orthopedic surgery for patients with severe cases of osteoarthritis. This surgery restores the range of motion in the knee joint and allows for pain-free movement. Advancements in medical techniques used in the surgical zone and implant technology, as well as the management of operations and administration for around two decades prior, have hugely improved surgical outcomes for patients. In this study, advancements in TKA were examined through exploring aspects such as robotic surgery, new implants and materials, minimally invasive surgery, and post-surgery rehabilitation. This paper entails a review of the peer-reviewed literature published between 2005 and 2025 in the PubMed and Google Scholar databases. For predictors, we incorporated clinical relevance together with methodological soundness and relation to review questions to select relevant research articles. We used the PRISMA flowchart to illustrate the article selection system in its entirety. Since robotic surgical and navigation systems have been implemented, surgical accuracy has improved, there is an increased possibility of ensuring alignment, and the use of cementless and 3D-printed implants has increased, offering durable long-term fixation features. The trend in the current literature is that minimally invasive knee surgery (MIS) techniques reduce permanent pain after surgery and length of hospital stays for patients, though the long-term impact still needs to be established. There is various evidence outlining that the enhanced recovery after surgery (ERAS) protocols show positive results in terms of functional recovery and patient satisfaction. The integration of these new advancements enhances TKA surgeries and translates them into ‘need of patient’ procedures, ensuring improved results and increases in patient satisfaction. The aim of this study was to perform a comprehensive analysis of the existing literature regarding TKA advancement studies to identify current gaps and problems.

## 1. Introduction

TKA, also commonly referred to as knee replacement, is a surgical technique carried out with the aim of reconstructing the knee joint of patients with severe osteoarthritis to eliminate pain and enhance movement. TKA restores the proper alignment, stability, and motion of the knee joint by replacing the worn-out surfaces of the femur and tibia as well as the patella. Total knee arthroplasty is one of the most reliable orthopedic surgical operations prevalent in the global community. The medical process of developing artificial knees started in the 1950s and 1960s, during which hinge-type implants were invented; these limited the anatomical movement of the knee joint, providing worse results compared to the types that were developed later. During the 1970s, total condylar knee designs were made, and these developments aimed to enhance prosthetic durability and mechanical performance. Progress made in the implant materials used, the use of new techniques in implant placement, and patient care processes has led to better treatment outcomes since the 1970s. TKA is among the most common surgeries carried out to relieve arthritis that orthopedic surgeons perform millions of times annually across the globe.

The worldwide need for knee arthroplasty procedures is increasing rapidly because of global aging populations and due to the fact that the percentages of patients with overweight and active-joint disease continue to grow. The combination of the demand for improvements in patient satisfaction, quicker recovery, and durable results created substantial incentives for TKA developments. Multiple technological and clinical improvements have been introduced throughout the last few decades that are able to maximize procedure precision and shorten recovery periods, allowing for these surgeries to better respond to individual patient needs. The medical field is also implementing robotic-assisted navigation systems with cementless and 3D-printed implants in addition to minimally invasive surgical approaches and improved rehabilitation guidelines. This review combines and provides a detailed evaluation of the most important breakthroughs in TKA over the last twenty years. This paper evaluates the recent literature to demonstrate clinical results stemming from modern total knee replacement innovations to generate a complete understanding for orthopedic surgeons and healthcare stakeholders regarding surgical landscape transformations.

## 2. Materials and Methods

This research compiled and assessed the most essential TKA innovations spanning from 2005 to 2025 through examinations of procedural development, implant innovations, and postoperative care plans. The authors conducted a search of the literature included in PubMed, Google Scholar, and Cochrane Library academic databases. We selected these databases because they provide extensive clinical and surgical research content. Medical Subject Heading terms joined with appropriate keyword terms enabled us to identify relevant advancements in TKA. The selected terms consisted of “Total Knee Arthroplasty”, “TKA”, “Robotic TKA”, “Cementless implants”, “3D-printed knee implants”, “Minimally Invasive Knee Surgery”, “Rehabilitation”, and “ERAS”. The search terms were linked using “AND” and “OR” Boolean operators to achieve precise results.

Sets of strict inclusion and exclusion criteria were set to ensure that we only selected high-quality research materials. The inclusion criteria for the studies were as follows:

Published between January 2005 and March 2025;
Involved human subjects;
The research material was written in the English language;
Published in peer-reviewed journals.
The exclusion criteria for the studies were as follows:
Involved animal models or in vitro experiments;
The available documents were written in languages other than English;
Editorials, preprints, and commentaries together with peer-reviewed publications.

The initial search yielded 1342 articles. The research resulted in 1180 distinct records that required screening. The evaluation of titles, followed by abstract review, resulted in us excluding 950 studies that failed to meet the inclusion criteria. The evaluation process of 230 full-text articles confirmed their suitability for evaluation. A review of complete articles resulted in the removal of 162 studies: 45 did not meet peer review standards, 57 were written in a language other than English, and 60 conducted their research with animals or in test tubes. The final analysis incorporated 68 studies that remained after qualification screening.

**Figure 1:**
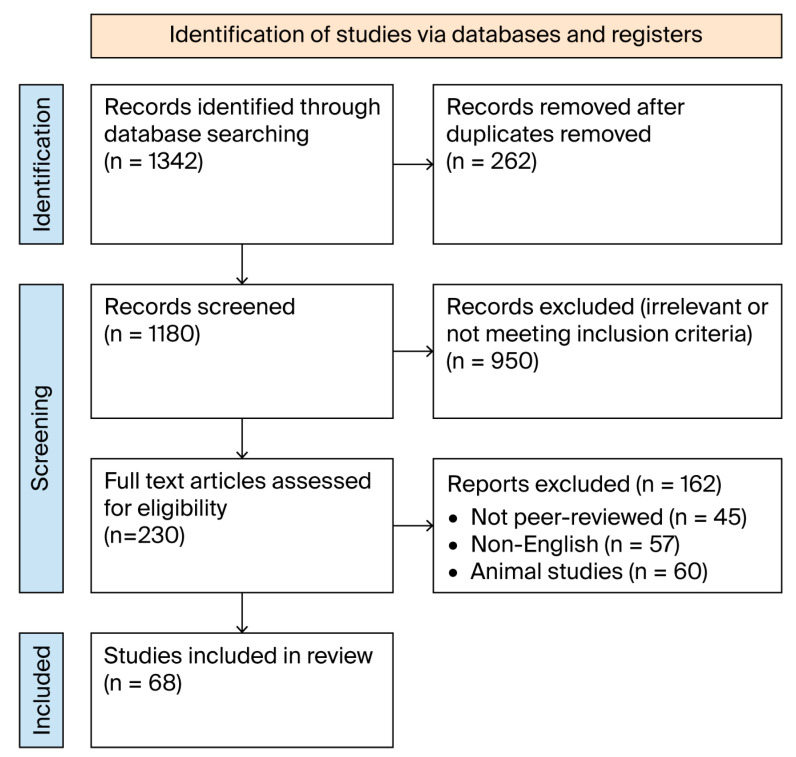
PRISMA flowchart illustrating the study selection process for the systematic review of advancements in total knee arthroplasty (2005–2025).

A PRISMA (Preferred Reporting Items for Systematic Reviews and Meta-Analyses) flow diagram describing the study selection phases is outlined in Figure 1, which also illustrates the final inclusion process [1].

## 3. Robotics and Navigation Systems

TKA benefits from robotic-assisted and computer-navigated systems, which provide better surgical accuracy alongside improved surgical planning techniques for individual patients. Multiple advanced robotic platforms have entered the market in the last twenty years, including MAKO and ROSA alongside NAVIO and VELYS systems. These systems help surgeons achieve precise bone resections and perfect implant positioning and balanced soft tissue structures, greatly affecting both the outcomes after surgery and prosthesis lifespan.

MAKO from Stryker stands as the most popular robotic system available for assisting TKA procedures. Knee and hip surgeries benefit from this system because they use CT imaging before surgery and measure bone position in real time, helping surgeons to make exact bone cuts and mold alignments with precise accuracy down to a single millimeter. The real-time ROSA (Zimmer Biomet) system functions without CT image requirements through the continuous analysis of anatomical references and continuous joint balancing. The NAVIO system from Smith & Nephew enables technical bone modifications through its handheld robotic interface; this system does not require imaging before surgery, thus allowing for greater intraoperative freedom. VELYS (DePuy Synthes) is a modern system that blends surgical navigation with robotic alignment controls to optimize placement while retaining the size and shape adapted to meet the surgeon’s needs [2,3,4].

The principal benefits of robotic-assisted TKA are that it allows physicians to achieve better component placement while decreasing procedural outliers and improving precise restoration of the mechanical axis [5,6]. Various research works show that robotic systems surpass conventional manual procedures by decreasing implant placement inconsistencies. The study conducted by Marchand et al. (2022) demonstrated that robotic TKA produced better alignment results through decreased complications in the coronal and sagittal planes [7]. Better management of soft tissues coupled with effective ligament balancing leads to satisfied patients who recover faster, with a potential increase in the lifespan of their prosthetic components [8,9].

**Figure 2:**
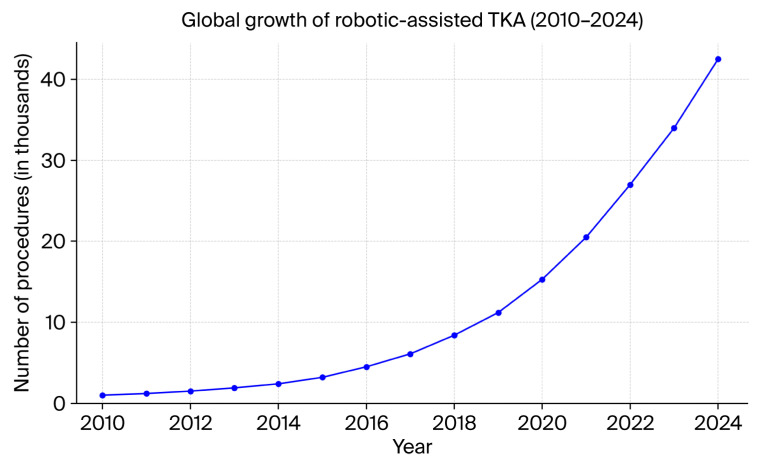
Chart illustrating global growth of robotic-assisted TKA over the last 15 years.

The current evidence shows positive results from robotic TKA, but there are still certain boundary limitations associated with the technology. The main drawback is related to cost because robotic systems require substantial startup investments, along with continuous maintenance and single-use equipment expenses for each procedure. The associated expenses restrict smaller or resource-limited institutions from obtaining these systems. Surgeons need to have completed an intense learning process before performing robotic TKA procedures. Treatment applicability requires surgical doctors to finish their training before being able to operate these systems effectively; there are generally longer procedure times when doctors first begin to adopt the use of these systems. Studies have revealed that robotic TKA produces outcomes equivalent to manual TKA for medium- and long-term assessments of implant survivorship alongside pain scores, thus rendering a need to scrutinize the economic benefits. The implementation of robotic systems results in better postoperative precision, but more high-quality, long-term research is essential to define their advantages regarding implant durability and patient functionality and satisfaction [10]. Orthopedic centers globally are adopting robotic platforms at an increasing rate as they move towards personalized joint replacement solutions that use technology assistance, as shown in Figure 2 [11].

## 4. Implant Design and Materials

The success and long-term performance of total knee arthroplasty (TKA) heavily depend on implant design and biomaterial engineering principles. Over the last 20 years, significant progress has been made regarding implant materials, fixation techniques, and customized prosthetic design. Technology developers have focused on improving the stability of implants while simultaneously decreasing wear rates and arbitrating individual patient skeletal structures. Polymethyl methacrylate (PMMA) bone cement has remained the established fixation technique for TKA since its introduction many years ago. The use of cemented implants allows for both quick initial joint stabilization and accurate predictions; many studies have also documented their longevity. However, alternative materials have emerged due to patient safety concerns, including aseptic loosening with prolonged use and cement degradation [12].

Today’s younger and more active patients show strong interest in cementless implants because these devices rely on bone ingrowth into porous surfaces for biological fixation. Cementless implants use trabecular metal or titanium foam porous coatings throughout their surface to help bones naturally integrate with the implant. Recent research investigating cementless TKA has found positive intermediate and extended results that demonstrate fewer cases of aseptic loosening and improved bone health retention [13,14]. The primary issues with older cementless systems have been resolved with present-day versions that show performance levels equivalent to cemented systems when utilized in high-quality bones [15].

The adoption of 3D printing techniques to make implants has resulted in major progress being made by combining precise surface designs with controlled pore structures while enhancing implant compatibility. The surfaces mimic the structure of trabecular bone tissue, thus enhancing bone connection to ensure stability and long-term attachment. Facilities that take preoperative images using 3D printing allow physicians to design implants specifically for a patient’s anatomy, which produces better alignment and personalized joint movement [16,17]. The advancements in implant design now focus on creating formats which better serve the patient population and their required functionalities. The medical field has developed gender-specific implants which meet the anatomical needs of male and female knees through modifications of femoral contours and Q-angle adjustments. Studies have produced varying results regarding patient benefits; there is an indication that female patients receive improved benefits from better-fitting implants as they support the movement of the patellar. The use of patient-matched instrumentation (PMI) combined with customized cutting guides based on MRI or CT data works to increase surgical accuracy and prosthesis placement, though more analysis is required in terms of their economic value [18,19].

Wear resistance plays an essential role in determining the performance of implants throughout their lifetime. Highly cross-linked polyethylene (HXLPE) has become the dominant choice for tibial insert applications since it surpasses conventional polyethylene in terms of its wear resistance properties. This new material decreases the occurrence of osteolysis, which often leads to the need for most revision surgical procedures [20,21,22].

## 5. Minimally Invasive Surgery

MIS techniques offer reduced soft tissue damage together with faster postoperative recovery and shorter hospital stay durations. TKA benefits from MIS since the procedure is performed through smaller incisions and there is minimal extensor mechanism disturbance. Early functional outcomes show that these procedures have no negative impact on prosthetic alignment or stability in the long-term [23,24]. Medical practitioners have created three fundamental MIS methods for total knee arthroplasty: the subvastus, midvastus, and quadriceps-sparing approaches. This latter method allows for access to the joint to be gained under the vastus medialis muscle without requiring quadriceps tendon splitting to maintain muscle quality. This type of surgical approach suits patients who enter the operation with solid movement ranges and minor joint malformation. The midvastus approach provides better visualization by cutting part of the vastus medialis but maintaining the important function of the quadriceps mechanism. The quadriceps-sparing technique provides the most minimally invasive solution since it keeps the quadriceps tendon intact while utilizing precise dissection to reach the joint capsule [25,26].

**Figure 3:**
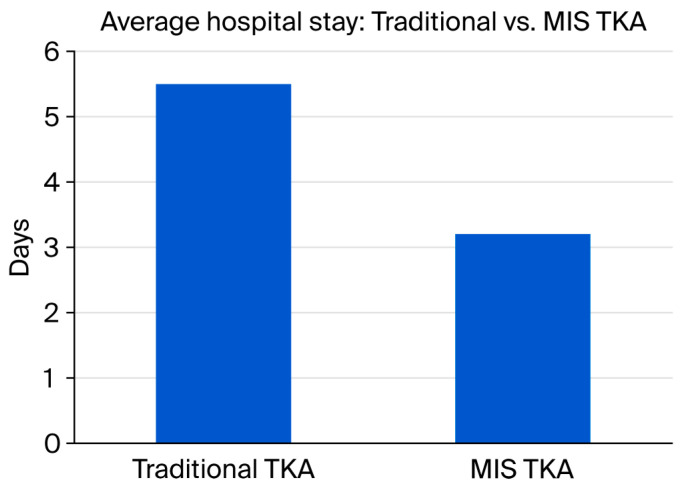
Comparison of average hospital stay duration (in days) between traditional TKA and minimally invasive surgery (MIS) techniques, showing reduced recovery time in MIS approaches.

Studies indicate that MIS procedures provide patients with smaller surgical scarring and reduced blood loss during operations, together with decreased postoperative pain intensity, resulting in better mobility and reduced hospital stay duration. Speedy recovery outcomes tend to benefit patients undergoing MIS-TKA since such techniques lead to better knee flexion along with improved muscle strength and gait function (Figure 3). Studies indicate that patients who undergo these procedures do not need to consume as many postoperative narcotics following surgery, and they also experience higher satisfaction during the first weeks after the operation. However, the medical community presents inconsistent findings regarding MIS use in total knee arthroplasty procedures. The main issue with MIS involves its potential to diminish operative views of essential surgical landmarks, which could lead to implant placement inadequacies or improper ligament tension. The lack of proper surgical exposure creates increased probabilities for patients to develop alignment problems, for the wrong implant size to be selected, and component loosening at the early stages after surgery. A significant number of research studies indicate both technical complications and a trend of extended operative durations during surgeons’ learning phase. A subset of patients, such as those suffering from extreme deformities, obesity, or those whose surgical history requires full exposure for proper access to implants and surgical elements, cannot benefit from MIS procedures [27].

Research now indicates that MIS-TKA demonstrates success in terms of safety, implant alignment, and long-term results when surgeons select suitable patients and gain proper experience. Review studies along with meta-analyses confirm that MIS-TKA and standard TKA provide equivalent operation survival rates and complication rates, but patients undergoing MIS recover more quickly in the first few months. Studies show that MIS procedures enable hospitals to reduce patient stay duration and also allow patients to go directly home after leaving the hospital instead of rehabilitation facilities. The utilization of these variables leads to healthcare cost reductions, especially when utilized in enhanced recovery protocols or outpatient TKA programs. Short-term benefits in patient recovery and satisfaction emerge from applying MIS techniques in TKA procedures. The long-term success of these procedures depends heavily on surgical expertise combined with suitable patient criteria and continuous monitoring for safe implant durability [28,29,30].

## 6. Enhanced Recovery and Rehabilitation Protocols

Today, ERAS protocols serve as an essential component of TKA that aims to achieve reduced complications as well as shorter hospital durations and satisfied patients. This model combines evidence-based multi-dimensional techniques throughout preoperative care and surgical operations for optimal patient recovery. TKA procedures using ERAS pathways contain multiple components, consisting of patient education, carbohydrate loading, regional anesthesia, opioid-sparing analgesia, as well as early mobilization and standardized discharge planning [31,32]. These surgical protocols offer two main benefits: they prevent surgery-induced stress and lower inflammatory response and promote the swift return of function. The current literature shows that ERAS works well in orthopedic surgery as it shortens hospital stays and reduces patients’ need to return to hospitals, providing better pain management while maintaining safety. Research into prehabilitation strategies has become increasingly popular because this involves preoperative physical and psychological improvements to optimize surgical outcomes. The recovery process includes strength exercises and heart condition training combined with nutrition advice and emotional preparation in the weeks before surgery. Patients who participate in preoperative preparation achieve better postoperative mobility, reduce their hospital stay duration, and display increased surgical readiness. There is a need to handle multiple health conditions, such as diabetes control together with smoking prevention and anemia therapeutic efforts, because these strides help minimize surgical complications [33].

Outpatient TKA represents a pivotal health service advancement because surgeons now perform total knee replacements where patients can be discharged on the same day as surgery. Physicians can use this procedure on patients who maintain a good medical condition along with supportive networks and demonstrate high independence in their daily activities. Scientific studies prove that outpatient TKA is safe and it delivers equally favorable or greater results than standard hospital-based procedures. Furthermore, healthcare costs and hospital-acquired infections decrease, and patients show increased satisfaction along with these benefits. The practice of TKA rehabilitation receives transformative power from modern technological developments. The use of medical technology through mobile applications, tele-rehabilitation systems, and wearable sensors provides remote patient monitoring of therapy outcomes, exercise tracking, and functional recovery monitoring. Doctors can use wearable technology to measure movement ranges, walking symmetry, and total step counts, allowing for therapeutic customization with early medical interventions in the case of treatment deviating from the expected outcomes. Digital rehabilitation tools provide maximum benefit to areas which lack adequate access to traditional therapy services in rural or underserved populations [34,35,36,37,38].

The medical community now utilizes patient-reported outcome measures (PROMs) together with functional scores for measuring how well recovery protocols work. The Knee Society Score (KSS), Oxford Knee Score (OKS), and Western Ontario and McMaster Universities Osteoarthritis Index (WOMAC) allow clinicians to measure how patients experience pain relief improvements together with their satisfaction levels. Patient outcomes from PROMs show that those participating in ERAS with prehabilitation programs achieve superior functional capacity during early recovery and greater treatment satisfaction when compared to traditional care methods [39,40,41,42].

## 7. Discussion

TKA is being continuously developed through modern innovations that represent improvements in surgical outcomes and accelerated recovery processes with better patient satisfaction results. In the field of TKA, doctors have integrated robotics together with MIS, cementless implants, and ERAS protocols. Multiple studies have demonstrated promising outcomes from these innovations in TKA, yet several studies present contradictory results that challenge these advances.

### 7.1. Robotics

#### 7.1.1. Supporting Studies

Research strongly supports robotic-assisted surgery in TKA because it delivers higher precision for implant placement. Robotic systems, including the MAKO and ROSA platforms, achieve better femoral and tibial alignment according to Wainwright, Jared D et al. Other studies show that robotic aid reduces surgical malalignment, leading to better treatment results, including faster patient recovery and a decreased need for revision procedures [43].

#### 7.1.2. Opposing Studies

According to Kirchner et al. (2024), the long-term benefits of robotic-assisted TKA for implant survival and functional outcomes remain inconsistent [44]. There are other studies that show no clinical benefit in augmenting TKA with a robot [45,46,47]. Further, large implementation barriers exist because of the initial expenses along with the substantial training requirements that surgeons must meet to use these systems, particularly apparent in regions with limited healthcare funding.

### 7.2. MIS

#### 7.2.1. Supporting Studies

Patients who have undergone MIS required smaller cuts to be made in their skin, experienced decreased postoperative discomfort and reduced bleeding amounts, and needed to stay in hospital for less time before their recovery began. Patients who received MIS treatment experienced better satisfaction rates because of the smaller scarring benefits [48].

#### 7.2.2. Opposing Studies

Many studies have identified problems caused by MIS methods when surgical staff encounter reduced visibility of important anatomical structures, since these methods make it harder for surgeons to set components correctly or maintain proper ligament balance [49,50,51]. Due to a lack of experience during the learning process, many surgeons require more time during surgery to perform these operations, which results in higher complication rates, especially during their first cases [52].

### 7.3. Implant Design and Materials

#### 7.3.1. Supporting Studies

Cementless implants have grown popular for active patients under the age of 50 because they provide both better bone integration and enhanced bone maintenance. Wang et al. (2020) demonstrated that cementless implants with porous coatings made of trabecular metal or titanium foam produce stronger fixation [53]. Cementless implants allow for long-term durability because they resist cement degradation throughout time [54,55].

#### 7.3.2. Opposing Studies

One research team in their study warns that cementless implants will not work effectively for patients who have poor bone quality. When applied to elderly patients or individuals with osteoporosis, implant fixation without cement might extend the time needed for bone integration and potentially raise the chance of implant failure [56]. Other studies show that cementless implant surgery may lead to increased aseptic loosening and no differences in clinical outcome [14,57,58,59].

### 7.4. ERAS Protocols

#### 7.4.1. Supporting Studies

The enhanced recovery after surgery (ERAS) protocol uses multiple strategic intervention techniques to maximize postoperative outcomes by educating patients, supporting nutritional needs before surgery, using pain management methods that do not include opioids, along with encouraging early rehabilitation. The implementation of ERAS pathways produced two strong results by shortening hospitalization times and creating better functional outcomes.

Patients who received care through the ERAS protocol experienced both diminished pain symptoms and a decreased need for opioid medications in the postoperative phase [60].

#### 7.4.2. Opposing Studies

Practitioners should exercise caution because, according to Wang et al. (2023), ERAS protocols may not match the needs of diverse patient groups [61]. Patients who have obesity or diabetes in conjunction with other comorbidities often do not receive similar advantages regarding recovery duration and complication occurrence. The high financial expenses associated with implementing ERAS protocols for complex surgical patients prompted evaluations about their economic value [62,63,64].

### 7.5. Synthesis of Literature: What Is Widely Accepted, Debated, or Still Under Research

These innovative approaches to TKA treatment yield inconsistent results in the available evidence (Table 1). Despite widespread recognition of the advanced precision robotic-assisted surgery provides for surgical teams, robotics’ overall value to healthcare presents long-term assessment challenges [65]. Most surgeons advocate for cementless implants with advanced porous coatings for young adult patients who perform physical activities despite ongoing concerns regarding their application for elderly people with poor bone quality [66]. The short-term benefits of MIS approaches for surgery recovery and pain relief are evident, yet doubts exist about potential consequences for both visualization and alignment deformation during these procedures [67]. The widespread implementation of ERAS protocols exists because they shorten hospital stays and improve recovery success, but healthcare providers need to be aware of who should undergo this treatment, and additional research is needed to assess high-risk patient outcomes.

## 8. Challenges and Future Directions

Many obstacles continue to prevent the general acceptance of the abovementioned innovations in TKA, despite encouraging developments being published. The main obstacle for the adoption of high-tech systems, including robotic-assisted surgery and patient-specific implants, is cost-related barriers. Large-scale investment in combination with maintenance costs and qualified staff training is beyond the financial capabilities of healthcare facilities operating under budget constraints. There is a lack of facilities available to support innovative medical equipment like advanced imaging systems for 3D modeling or robotic systems within the healthcare environment.

Training is another significant barrier. Advanced MIS techniques as well as robotic system operations require specific training for surgeons, which could include a potentially challenging learning process with newer medical technology. Without adequate training, the risk of complications and suboptimal outcomes increases. Research gaps exist in the medical literature regarding the permanent effects that newer surgical technologies have on patient outcomes, especially for cementless implants and robotic-assisted surgery. The evaluation of short-term benefits has been confirmed, yet the long-term effects on implant survival rates together with revision cases and patient satisfaction levels remain without definitive answers.

The upcoming advancements in total knee arthroplasty involve artificial intelligence-driven surgical planning systems for individual medical procedures and customized three-dimensional modeling creation and biomimetic implant designs, which increase natural tissue bonding [68]. Such technological advances will hopefully lead to better precision and improved patient outcomes. The implementation of innovative solutions requires continuous attention to equity in order to make them accessible to patients. Medical organizations face an ongoing difficulty of guaranteeing that healthcare benefits reach each patient regardless of their economic position or residency area, especially in times of crisis, as it was during the COVID-19 pandemic [69].

## 9. Conclusions

Several advancements in TKA over the last two decades, such as surgical robots along with MIS, cementless hardware, and ERAS protocols, have revolutionized TKA by improving surgical precision while reducing recovery time and enhancing patient satisfaction. These new techniques have transformed the field of TKA by delivering better customized and optimized knee replacement solutions. The research on their extended benefits remains in an ambiguous state; thus, future studies are needed to determine their genuine impact on implant survival and functional results together with cost-effectiveness levels. Medical professionals should apply evidence-based methods in the assimilation of these cutting-edge technologies. Future advancements in TKA may further extend to patient-specific solutions for rare conditions such as ochronotic arthropathy, where successful joint replacement has been reported [70].

## Figures and Tables

**Table 1:** Comparison of evidence strength and clinical applicability for key TKA innovations.

TKA Innovation	Evidence Strength	Clinical Benefit	Implementation Barriers
Robotic-Assisted Surgery	High (Multiple RCTs, meta-analyses)	Improved precision, soft tissue balancing	High cost, long learning curve
Cementless Implants	Moderate-High (Long-term observational, meta-analyses)	Durable fixation in younger patients	Not ideal for osteoporotic bone
Minimally Invasive Surgery	Moderate (Mixed results from RCTs)	Faster recovery, less pain early post-op	Reduced visualization, higher technical skill required
ERAS Protocols	High (Systematic reviews, meta-analyses)	Reduced LOS, better pain control	Requires institutional coordination, not ideal for all patient groups

## Data Availability

The articles used in this review can be found on the PubMed and Google Scholar databases.

## Abbreviations

The following abbreviations are used in this manuscript:

TKA	Total Knee Arthroplasty
PRISMA	Preferred Reporting Items for Systematic Reviews and Meta-Analyses
MIS	Minimally Invasive Surgery
ERAS	Early Rehabilitation After Surgery
CT	Computer Tomography
MRI	Magnetic Resonance Imaging
HXLPE	Highly Cross-Linked Polyethylene
PMMA	Polymethyl Methacrylate
